# Current status of BAFF targeting immunotherapy in B-cell neoplasm

**DOI:** 10.1007/s10147-024-02611-2

**Published:** 2024-09-02

**Authors:** Nami Tagami, Junichiro Yuda, Yasuyuki Goto

**Affiliations:** 1 Laboratory of Molecular Immunology, Graduate School of Agricultural and Life Sciences, The University of Tokyo, 1-1-1 Yayoi, Bunkyo-ku, Tokyo, 113-8657 Japan; 2 Department of Hematology, National Cancer Center Hospital East, 6-5-1 Kashiwanoha, Kashiwa, Chiba 277-8577 Japan

**Keywords:** BAFF, B-cell proliferation, Hematological malignancy

## Abstract

B-cell activating factor belonging to the TNF family (BAFF), also known as B-lymphocyte stimulator (BLyS), plays a crucial role in B-cell development. It has multiple receptors, including BCMA, TACI, and BAFF-R, with diverse roles in different cell types. BAFF induces B-cell proliferation and immunoglobulin secretion, and acts as a survival factor for immature, naive, and activated B cells. Consequently, BAFF-deficient mice often show suppressed humoral responses, while BAFF-overexpressing mice show the higher number of mature B cells and may develop autoimmune-like manifestations and B-cell lymphoproliferative diseases. Elevated BAFF levels are also associated with various hematological malignancies, and its expression correlates with disease progression in some cases. Therefore, BAFF-targeted therapies, such as belimumab, atacicept, and tabalumab, are being explored in clinical trials for conditions like chronic lymphocytic leukemia (CLL) and multiple myeloma. Belimumab, an anti-BAFF monoclonal antibody, is being investigated in combination with rituximab/venetoclax for CLL. Atacicept, a decoy receptor for BAFF and APRIL, showed tolerability in a phase 1b trial for CLL. Tabalumab, another monoclonal antibody targeting BAFF, did not demonstrate significant efficacy in a phase 2 study for relapsed/refractory multiple myeloma. BAFF ligand-based CAR-T cells are designed to target BAFF receptors and show promise in preclinical studies, particularly for B-cell malignancies. The review emphasizes the importance of understanding the roles of BAFF and its receptors in the microenvironment of hematologic malignancies. Targeting BAFF and its receptors presents potential therapeutic avenues, and ongoing clinical trials provide valuable insights.

## Introduction

### The role of BAFF

**Fig. 1:**
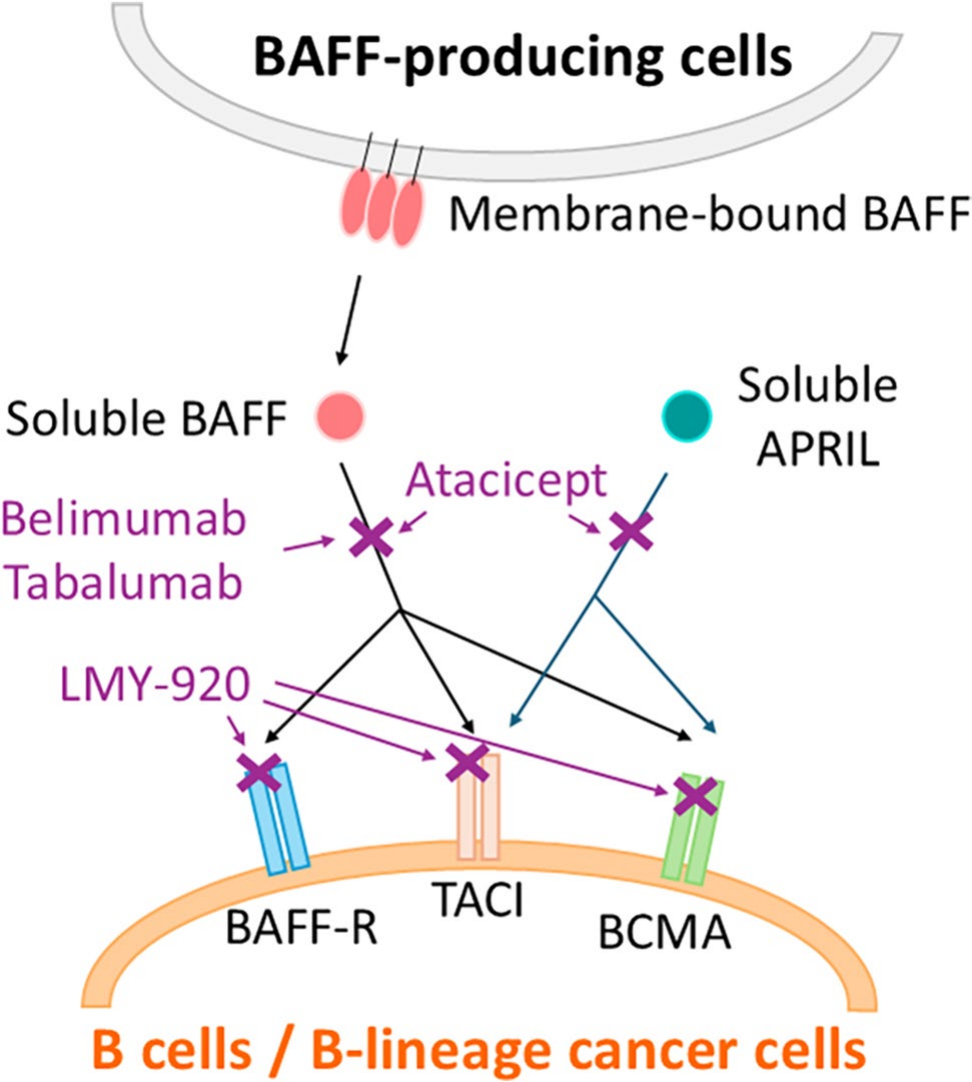
Interactions between BAFF receptors (BAFF-R, TACI, BCMA) and their ligands (BAFF, APRIL) and interventions targeting these molecules. BAFF can bind to all of BAFF-R, TACI, and BCMA, whereas APRIL can bind to TACI and BCMA. Because belimumab and tabalumab are monoclonal anti-BAFF antibodies, these will block BAFF-mediated signaling through BAFF-R, TACI, and BCMA. On the other hand, atacicept is a TACI-based decoy receptor and can inhibit both BAFF and APRIL. LMY-920 is a BAFF-CAR-T and then can target cells expressing any of the BAFF receptors

B-cell activating factor belonging to the TNF family (BAFF) is a critical molecule in B-cell development, known by various synonyms, such as B-lymphocyte stimulator (BLyS), zTNF4, TNF homologue that activates apoptosis, nuclear factor κB, c-Jun NH2-terminal kinase (THANK), TNF and apoptosis ligand-related leukocyte-expressed ligand 1 (TALL¬1), and TNFSF13B [1–5]. BAFF is expressed by monocytes, macrophages, dendritic cells, and lymphoid cells including B cells and activated T cells [2, 3, 6]. BAFF induces B-cell proliferation and immunoglobulin secretion, and is an important survival factor for immature, naive, and activated B cells [1, 2]. BAFF induces survival of a subset of splenic immature B cells known as transitional type 2 (T2) B cells. BAFF treatment allows T2 B cells to survive and differentiate into mature B cells in response to signals through the B-cell receptor (BCR) [7]. BAFF is found either on the cell surface as a type II transmembrane protein or as a soluble form after cleavage by a protease called Furin [2]. There are three separate receptors for BAFF; BCMA (B-cell maturation antigen), TACI [transmembrane activator and calcium-modulator and cyclophilin ligand (CAML) interactor], and BAFF-R (BAFF receptor; BR3) (Fig. 1) [1, 8]. The receptor-binding domain of BAFF is trimeric, and BAFF trimers can assemble into a BAFF 60-mer [8]. Interferon-γ (IFN-γ) and IFN-α both upregulate the expression of BAFF by monocytes and dendritic cells (DCs) [2, 8]. IL-10 also stimulates the secretion of BAFF by macrophages [1]. BAFF can also be expressed by T lymphocytes and promote T-cell activation and survival [9]. BAFF signaling in T cells and potential T-cell modulation in response to a BAFF-modified B-cell compartment may contribute significantly to inflammation and immunomodulation [9]. The amino-acid sequence of BAFF is related most closely to that of a proliferation-inducing ligand (APRIL), also known as TNFSF13A [10]. APRIL is expressed at a low level by normal lymphoid and myeloid cells and at a high level by a variety of human cancers [11]. APRIL binds to BCMA and TACI, but not to BAFF-R [12]. APRIL is cleaved intracellularly in the Golgi site prior to release and normally exists in a soluble form only, once outside of the cell of origin [8, 13, 14]. BAFF and APRIL are suggested to share several biological activities, indicating potential functional similarities [8].

Autocrine production of BAFF is emphasized in primary B-cell chronic lymphocytic leukemia (B-CLL) and myeloma cells [15, 16]. Significantly elevated levels of BAFF were observed in the blood samples of patients with B-CLL, diffuse large B-cell lymphoma (DLBCL), follicular lymphoma (FL), cutaneous T-cell lymphoma (CTCL), acute myeloid leukemia (AML), acute lymphoblastic leukemia (ALL), non-Hodgkin’s lymphoma (NHL), and multiple myeloma (MM), compared with healthy donors [16–27]. Patients with low baseline BAFF expression had significantly longer median progression-free survival (PFS) than those with high BAFF expression in patients with MM [28, 29], DLBCL [21], CTCL [17], and AML [30]. On the other hand, varied reports were found regarding the relationship between BAFF level and tumor response, and others reported no clear association in patients with MM [31], DLBCL [32], FL [33, 34], CLL [35], and AML [36]. This underscores the complex and context-dependent role of BAFF in hematologic malignancies, impacting both disease progression and treatment responses.

### BAFF-knockout/BAFF-transgenic mouse model

BAFF-knockout (BAFF-KO) mice show no apparent birth defect and grow to at least 6–8 months of age without unusual morbidity, and all major organs, including thymus, spleen, and lymph node, are present, although average spleen weights of BAFF-KO mice are significantly reduced [37, 38]. BAFF-KO mice have significantly fewer marginal zone and follicular B cells than wild-type animals [37]. The remaining B lymphocytes mostly exhibited staining like that of T1 transitional B cells [37]. Although these cells were normal in number, there were almost no cells of a T2 phenotype [37, 39].

BAFF-KO mice exhibit a deficiency in mature B cells and an impaired immune response, in contrast to BAFF-transgenic (BAFF-Tg) mice, which, due to their heightened production of BAFF, display increased numbers of mature B cells and effector T cells [8, 40]. There is a correlation between excess BAFF in BAFF-Tg mice and the development of autoimmunity, resembling systemic lupus erythematosus (SLE) in humans [41]. BAFF-Tg mice exhibit autoimmune-like manifestations, including the presence of high levels of rheumatoid factors, circulating immune complexes, anti-DNA autoantibodies, and immunoglobulin deposition in the kidneys [40]. A small percentage (3–5%) of BAFF-Tg mice spontaneously develop B-cell lymphoproliferative diseases during aging [42]. The development of B-cell lymphoproliferative diseases in BAFF-Tg mice appears to be linked to the action of tumor necrosis factor (TNF), as introducing TNF deficiency into a BAFF-Tg background increases the incidence of B-cell lymphoma [43]. Inefficient B-cell maturation in hematopoietic humanized mice is not attributed to suboptimal bioactivity of murine BAFF on human B cells [44]. These mouse models provide valuable insights into the intricate relationship between BAFF, immune function, and the development of autoimmune and lymphoproliferative diseases, shedding light on potential therapeutic targets and mechanisms underlying immune system regulation.

## Receptors for BAFF and induced signals

### BAFF receptor (BAFFR)

BLyS receptor 3 (BR-3; BAFF-R) is specific for BAFF and appears to be the principal receptor for BAFF-mediated mature B cell survival [45]. High levels of BAFF-R mRNA were detected in the spleen and lymph nodes, lower in the peripheral blood leukocytes and thymus, and little in the bone marrow or fetal liver [46]. BAFF-R is essential for survival and maturation of immature B cells [8]. BAFF‐R is also expressed on activated or memory T cells and BAFF plays important roles in T‐cell activation through BAFF-R [17, 47]. The best described signaling process from BAFF-R is the activation of the non-canonical NF-κB pathway. BAFF-R also transduces signals to several other pathways, including phosphoinositide 3-kinase (PI3-kinase) and ERK1/ERK2 kinases [8]. There are some reports regarding BAFF-R and B-cell malignancies. The majority of patients with DLBCL were positive for both BAFF and BAFF-R. The lack of BAFF-R expression might independently increase the risk of overall survival (OS) in patients with DLBCL [32, 48]. On the other hand, high expression of BAFF-R, but not BAFF, may be an independent risk factor for PFS and OS in FL [33]. Also, single nucleotide polymorphisms (SNPs) in *BAFF* and *BAFF-R* genes may be considered as potential CLL risk factors [49]. MM cells express little, if any, cell surface BAFF-R [50].

### Transmembrane activator and calcium-modulator and cyclophilin ligand interaction (TACI)

TACI, one of the TNF receptor family members, binds to BAFF and APRIL [4, 51, 52]. TACI is critical for T-cell-independent responses of B cells to type I and type II antigens, negative regulation of the size of the B-cell compartment, and class-switch recombination [8]. It is a potent activator of the NF-κB signaling pathway and can interact with TRAF2, 3, 5, and 6. TACI also promotes the differentiation and survival of plasma cells [53–56]. An allelic variation in the gene encoding for the TACI protein is associated with CLL susceptibility, suggesting a potential role for TACI in disease development [57, 58]. Tumor cells in CTCL express both BAFF and its receptors, BAFF‐R and TACI [17]. BAFF signaling via TACI promotes IL-10 production by CLL B cells in a mouse model and in CLL patients. Moreover, BAFF-mediated IL-10 production by normal and CLL B cells is mediated through TACI [59].

### B-cell maturation antigen (BCMA)

BCMA binds to BAFF and APRIL [60, 61]. It is a B-cell-specific receptor, not expressed on T cells, and is particularly found on plasmablasts [9]. BCMA promotes plasma-cell survival [8]. BCMA is highly expressed in plasma cells, which are the final terminally differentiated form of B cells. Its specificity to plasma cells makes it a promising therapeutic target for multiple myeloma, a type of cancer that affects plasma cells [62]. The specific signaling pathways of BCMA leading to plasmablast and/or plasma-cell survival have not been fully investigated. However, it is suggested that BCMA activates the classical NF-κB pathway, likely through its ability to bind members of the receptor-associated factor (TRAF) family. BCMA undergoes cleavage by the c-secretase and γ-secretase enzyme complexes. The c-secretase enzyme complex (GS) cleaves BCMA, releasing the extracellular domain and a truncated piece of the intramembranous part, resulting in soluble BCMA (sBCMA) [62]. γ-secretase directly cleaves BCMA without prior truncation by another protease [63].

BCMA is detected in the serum of untreated MM patients, with levels higher than those in patients with monoclonal gammopathy of undetermined significance (MGUS) and healthy subjects. Additionally, serum BCMA levels were found to be higher in patients with progressive disease compared to those with responsive disease [64]. sBCMA has been proposed as a potential biomarker to identify patients with MGUS and smoldering multiple myeloma (SMM) who are at an increased risk of progression to MM. sBCMA may serve as an independent indicator beyond established risk models [65].

Kaplan–Meier analysis revealed that serum BCMA levels above the median are predictive of shorter progression-free survival and OS in multiple myeloma patients [65]. Specifically, patients with elevated serum BCMA levels at the start of front-line or new salvage therapy experienced shorter progression-free survival [65]. sBCMA is suggested to sequester circulating BAFF, preventing it from performing its signaling role in stimulating normal B-cell and plasma-cell development. This process may lead to reduced polyclonal antibody levels in MM patients [66]. Plasma BCMA is highlighted as a promising prognostic and predictive indicator for patients with CLL [67]. There is a mention of myeloma cells escaping BCMA-targeted chimeric antigen receptor T-cell (CAR-T) therapy by losing or reducing antigens. The remaining clones that escape CAR-T therapy may contribute to relapse in MM patients [68].

## Targeting BAFF in disease

BAFF-targeted therapies for hematopoietic malignancy are summarized in Table 1.

**Table 1:** BAFF-targeted therapies for hematopoietic malignancy

Drug name	Target	Phase	Condition or disease	Intervention	ClinicalTrials.gov Identifier
Belimumab	Anti-BAFF antibody	2	Relapsed or Refractory CLL	Belimumab–rituximab/venetoclax	NCT05069051
Tabalumab /LY2127399	Anti-BAFF antibody	2	Relapsed or Refractory MM	Tabalumab–Bortezomib–Dexamethasone (Bd) vs Bd	NCT01602224
		1	Relapsed or Refractory MM	Tabalumab–Bortezomib–Dexamethasone (Bd)	NCT01556438
		1	Relapsed or Refractory MM	Tabalumab	NCT00689507
Atacicept	TACI-Ig, BAFF/APRIL dual inhibitor	1	Relapsed or Refractory CLL	Atacicept	N/A
LMY-920	BAFF CAR-T cells	1	Relapsed or Refractory NHL	LMY-920	NCT05312801
		1	Relapsed or Refractory Myeloma	LMY-920	NCT05546723

### Belimumab in relapsed or refractory CLL

Belimumab is a human monoclonal antibody (IgG1, λ chain) that inhibits B-cell differentiation and survival by inhibiting the biologic activity of BAFF. BAFF plays a role in the survival and maturation of B cells, and inhibiting it can lead to the depletion of B-cell population [69]. FDA approved belimumab in 2011 as the first targeted biological treatment for SLE, and later, in 2020, it was approved as the first treatment for lupus nephritis [70, 71]. A phase II clinical trial is underway, exploring the use of belimumab in combination with rituximab/venetoclax for patients with refractory or relapsed CLL [72]. One identified mechanism of therapy resistance in CLL involves reduced sensitivity to rituximab-induced antibody-dependent cell-mediated cytotoxicity (ADCC) due to natural killer (NK) cell production of BAFF [72]. Additionally, recombinant human BAFF can reverse the cytotoxic effects of venetoclax, and this effect may be countered by the application of belimumab. The trial aims to remove BAFF from the CLL microenvironment, thereby enhancing the efficacy of rituximab/venetoclax treatment. The anticipated outcomes include achieving higher and earlier minimal residual disease (MRD) negativity rates and potentially allowing for abbreviated treatment.

### Atacicept in relapsed or refractory CLL

Atacicept is a recombinant, soluble fusion protein that acts as a “decoy receptor” for BAFF and APRIL. Atacicept binds to soluble APRIL, soluble BAFF, and membrane-bound BAFF. Atacicept failed to demonstrate a superior effect on disease activity compared to placebo in patients with multiple sclerosis (MS), optic neuritis, rheumatoid arthritis (RA), or SLE [73, 74]. A phase 1b clinical trial investigated the tolerability and biological activity of escalating doses of intravenously administered atacicept in 24 patients with refractory or relapsed CLL [75]. The trial results indicated that up to 27 mg/kg of atacicept administered intravenously was well tolerated in heavily pretreated patients with CLL. This information is valuable for informing future clinical trials involving atacicept in various B-cell disorder [75].

### Tabalumab in multiple myeloma

Tabalumab (LY2127399) is a fully human IgG4 monoclonal antibody designed to target and neutralize both soluble and membrane-bound BAFF. It is currently under investigation in clinical trials for several conditions, including RA, SLE, MM, MS, and end-stage renal disease [19, 28, 29, 31, 76]. In a double-blind, Phase 2 study, 220 patients with relapsed/refractory MM were randomly assigned to receive placebo, tabalumab 100 mg, or tabalumab 300 mg. All patients received treatment in combination with dexamethasone and subcutaneous bortezomib. The study did not observe significant intergroup differences among primary (median PFS) or secondary efficacy outcomes. This suggests that, in this particular study, tabalumab did not demonstrate a significant improvement in PFS compared to the placebo when used in combination with dexamethasone and bortezomib for relapsed/refractor MM [28].

### BAFF ligand-based chimeric antigen receptor (*CAR*)-T cells

CAR-T therapy involves genetically modifying a patient's T cells to express a receptor that targets specific antigens on cancer cells. In this case, BAFF ligand-based CAR-T cells are designed to target cancerous B cells. These CAR-T cells are engineered to target BAFF receptors, including BAFF-R, TACI, and BCMA [77, 78]. The rationale for this approach is the more limited expression of these receptors during B-cell development, making it a potentially more selective strategy to eliminate malignant B cells. BAFF ligand-based CAR-T cells have shown effectiveness in killing cells from various B-cell malignancies, including ALL, mantle cell lymphoma (MCL), and MM cells both in vitro and in vivo [77, 78]. A phase 1 clinical trial has been initiated to evaluate the safety and efficacy of BAFF ligand-based CAR-T cells in treating relapsed or refractory NHL patients, primarily focusing on MCL [79].

## Conclusion and future perspective

**Fig. 2:**
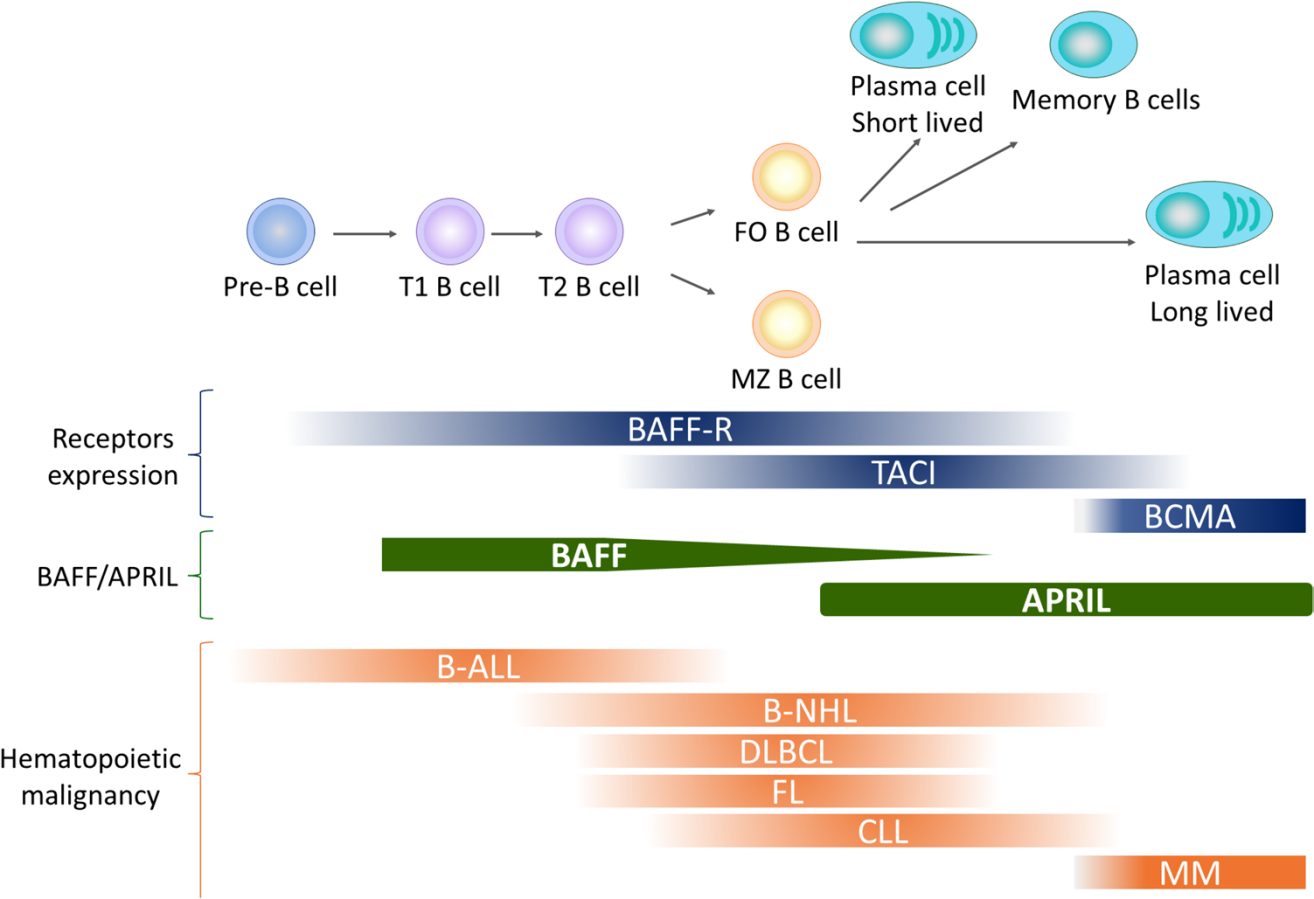
Expression of BAFF receptors and BAFF and APRIL-induced signals in B-cell development pathways. Related hematopoietic malignancy is shown by the presence of the respective receptors and ligand

This review is a comprehensive overview of the role of BAFF and its receptors in hematological malignancies, particularly in the context of B-cell differentiation, maturation, and the potential therapeutic implication. The expression pattern of BAFF receptors, such as BAFF-R and BCMA, varies depending on the stage of B-cell differentiation [80–84]. BAFF-R is expressed at the early stage of B-cell development, while BCMA is highly expressed in plasma cells, representing the terminally differentiated form of B cells [62]. BAFF has been shown to inhibit apoptosis of lymphoma, CLL, and MM cells in vitro [15, 18, 23, 28, 85]. The association of BAFF and its receptors in the tumor microenvironment of hematologic malignancies make them potential therapeutic targets. Additionally, treatments targeting BAFF, when used in combination with the existing therapies, have a low potential for reducing the efficacy of the existing treatments, while there is a possibility of obtaining synergistic effects (Fig. 1). Identifying hematopoietic malignancies highly dependent on BAFF and BAFF receptors is crucial for understanding the mechanisms of onset and progression of these malignancies. Hematologic malignancies encompass a range of cancers, including leukemias, lymphomas, and MM, arising from abnormal differentiation of hematopoietic stem cells (HSCs) in the bone marrow. Abnormalities in HSC differentiation result in a spectrum of disorders and malignancies [86]. Targeting antigens highly expressed on the cell surface at abnormal differentiation stages can be a therapeutic strategy for specific hematologic malignancies. The expression levels of receptors and ligands are believed to correlate with B-cell differentiation in hematologic malignancies (Fig. 2). Understanding the role of BAFF and its receptors in the microenvironment of hematologic malignancies is expected to elucidate the mechanisms of pathogenesis and progression of these malignancies. In summary, the interplay between BAFF, its receptors, and the immune responses in the context of hematologic malignancies provides valuable insights for potential therapeutic interventions and a deeper understanding of the underlying mechanisms of these diseases.

## Data Availability

Not applicable.
